# Cycle Threshold Values of SARS-CoV-2 RT-PCR during Outbreaks in Nursing Homes: A Retrospective Cohort Study

**DOI:** 10.3390/epidemiologia5040046

**Published:** 2024-10-16

**Authors:** Juan Carlos Gascó-Laborda, Maria Gil-Fortuño, Maria Dolores Tirado-Balaguer, Noemi Meseguer-Ferrer, Oihana Sabalza-Baztán, Óscar Pérez-Olaso, Iris Gómez-Alfaro, Sandrine Poujois-Gisbert, Noelia Hernández-Pérez, Lledó Lluch-Bacas, Viorica Rusen, Alberto Arnedo-Pena, Juan Bautista Bellido-Blasco

**Affiliations:** 1 Epidemiology Division, Public Health Center, 12003 Castelló de la Plana, Spain; gasco_jua@gva.es (J.C.G.-L.); meseguer_noe@gva.es (N.M.-F.); lluch_lle@gva.es (L.L.-B.); rusen_vio@gva.es (V.R.); bellido_jua@gva.es (J.B.B.-B.); 2 Microbiology Laboratory, Universitary Hospital de la Plana, 12540 Vila-Real, Spain; gil_marfor@gva.es (M.G.-F.); perez_oscola@gva.es (Ó.P.-O.); poujois_san@gva.es (S.P.-G.); hernandez_noeper@gva.es (N.H.-P.); 3 Microbiology Laboratory, Universitary General Hospital, 12004 Castelló de la Plana, Spain; tirado_dolbal@gva.es (M.D.T.-B.); sabalza_oih@gva.es (O.S.-B.); gomez_iri@gva.es (I.G.-A.); 4 Department Health Sciences, Public University Navarra, 31006 Pamplona, Spain; 5 Public Health and Epidemiology, Centro Investigación Biomédica en Red España (CIBERESP), 28029 Madrid, Spain; 6 Department of Epidemiology, School of Medicine, Jaume I University, 12006 Castelló de la Plana, Spain

**Keywords:** SARS-CoV-2, PCR, cycle threshold, infections, nursing homes, outbreaks, retrospective cohort

## Abstract

Backgound/Objectives: Cycle threshold (Ct) values of SARS-CoV-2 real-time reverse transcriptase-polymerase chain reaction (RT-PCR) tests are associated with infectivity and viral load, and they could be an aid in forecasting the evolution of SARS-CoV-2 outbreaks. The objective was to know the Ct values related to the incidence and reinfection of SARS-CoV-2 in successive outbreaks, which took place in nursing homes in Castellon (Spain) during 2020–2022, and to test its usefulness as an instrument of epidemic surveillance in nursing homes. Methods: a retrospective cohort design with Poisson regression and multinomial logistic regression were used. Results: We studied four nursing home SARS-CoV-2 outbreaks, and the average infection rate, reinfection rate, and case fatality were 72.7%, 19.9%, and 5.5%, respectively; 98.9% of residents were vaccinated with three doses of a mRNA SARS-CoV-2 vaccine. Ct values for first infections and reinfections were 27.1 ± 6.6 and 31.9 ± 5.4 (*p* = 0.000). Considering Ct values ≥ 30 versus <30, residents with reinfections had Ct values higher than residents with a first infection, an adjusted relative risk of 1.66 (95% Confidence interval 1.10–2.51). A sensitivity analysis confirmed these results. Conclusions: Reinfection and SARS-CoV-2 vaccination (hybrid immunity) could protect against severe disease better than vaccination alone. High Ct values suggest lower transmission and severity. Its value can be useful for surveillance and forecasting future SARS-CoV-2 epidemics.

## 1. Introduction

Cycle threshold (Ct) values of the real-time reverse transcriptase-polymerase chain reaction (RT-PCR) have been considered in the diagnosis, follow-up, and infectivity of SARS-CoV-2 in many studies [1,2,3,4,5,6,7,8,9]. However, the intrinsic values of Ct in SARS-CoV-2 outbreaks in nursing homes have been less studied [10,11,12], and its utility as a tool for forecasting epidemics and new variants has been only described in the general population [13,14,15]. Nursing homes were crucial points of the SARS-CoV-2 pandemic, considering the devastating impact on the elderly population residents, who had a high risk of infection, and many of this population presented immune deficiencies that increased the persistence and viral SARS-CoV-2 load [16,17].

Preventive and control measures to stop the transmission of the virus can be made considering Ct values, which are correlated with duration, infectivity, and viral load of infected patients [18,19]. In addition, SARS-CoV-2 vaccines can increase Ct after one or two doses, producing a lower viral load and decreasing virus transmission [20,21].

However, the use of Ct values in clinical and epidemiological contexts has controversy; Ct values are not standardized, and their limits have been the subject of some discussion, but more than 34 cycles are considered negative [22,23,24]. On the other hand, considering Ct values higher than 35 as negative to transmit the virus would be a limitation, given that it has been estimated that around 4.7 and 8.3% of patients with Ct values higher than 35 are infectious [18,25]. In addition, some research has indicated that Ct values are not associated with the clinical history of the disease and are only weakly associated with symptomatology at the time of the test [26]. Routine reporting of Ct values is not recommended after considering several critical points, including collected samples, transport, analytic methods, and international standardization [27,28]. However, at a population level, Ct values could be used to know the evolution of the epidemic with a standardized assay [15].

Our hypothesis was that SARS-CoV-2 reinfection plus SARS-CoV-2 vaccine (hybrid immunity) could offer better protection against severe disease and viral transmission, and this could be measured by Ct values. The objective of this study was to estimate the Ct values related to the incidence and reinfection of SARS-CoV-2 in four outbreaks that took place in nursing homes in Castellon (Spain) and to test Ct values as an instrument of epidemic surveillance in these facilities.

## 2. Materials and Methods

### 2.1. Retrospective Cohort Study

The study population corresponds to residents in four nursing homes in the Health Department of Castellon, Valencia Community (Spain), during the period 2020–2022, where COVID-19 outbreaks took place. The Epidemiology Division of the Public Health Center of Castelló de la Plana implemented the actions to control these outbreaks in order to break SARS-CoV-2 transmissions and prevent new cases. From the information collected, a retrospective cohort design was used to address the objective of the study. In total, four outbreaks occurred in the four studied nursing homes from February 2021 to April 2022. The inclusion criteria of residents were to suffer a SARS-CoV-2 infection or reinfection during the study period, be tested by PCR, and have a Ct value. The exclusion criteria were that no one suffered an SARS-CoV-2 infection or was not to be tested by PCR, and no one had a Ct value.

All SARS-CoV-2 cases had a laboratory confirmation test by a positive RT-PCR carried out by the Microbiology Services of the University General Hospital in Castelló de la Plana and the University Hospital of La Plana in Vila-real. For the RT-PCR we were using Roche Lightmix Modular SARS-CoV-2 (Roche-TIB MOLBIOL D-12103, Berlin, Germany) [29] the VIASURE SARS-CoV-2 Real Time PCR Detection Kit (CerTest Biotec S.L, Zaragoza, Spain), Abbott Real-Time SARS-CoV-2 (Abbott Laboratory, Chicago, IL, USA), and Argene SARS-CoV-2 R-Gene (Biomérieux SA, Marcy-l'Étoile, France). Less than 30 Ct values were considered positive following the official protocol of health authorities [30]

We defined reinfection as a new SARS-CoV-2 infection more than 60 days after the previous SARS-CoV-2 infection and confirmed by PCR test or a rapid antigen test (RAT) [31]. The first infection must have been confirmed by PCR, RAT, or positive anti-nucleocapsid IgG antibodies.

### 2.2. Statistical Analysis

Comparisons of qualitative and quantitative variables were made with the Chi-squared test, Fisher’s test, and Kruskall–Wallis test. First, we used Ct values as a quantitative dependent variable and SARS-CoV-2 reinfection as an explicative variable. One-way analysis of variance (ANOVA) and robust bivariate and multivariable linear regressions were employed in the analysis, as other studies have been utilized [32,33,34,35]. Second, we used the Ct value as a dependent variable, considering Ct value ≥ 3.0 as value 1 and Ct value < 30 as value 0. SARS-CoV-2 reinfection was considered a predictor variable, and Poisson regression was employed to calculate the crude relative risk (cRR) and adjusted relative risk (aRR) with a 95% confidence interval (CI). For all the multivariable models, age, sex, nursing home, and elapsed time from the first SARS-CoV-2 outbreak were included as confounding factors following the Directed Acyclic Graphs (DAGs) method in order to measure the relationship between reinfection (exposure) and Ct values (outcome). We used the DAGitty program version 3.1 [36], and the employment of this methodology is habitual [37,38]. For the implementation of multivariable models and the rest of the statistical analysis, we used the Stata^®^ program 14 version 2, a program employed in epidemiological studies [39,40].

To confirm the results of the first analysis, following a suggestion of the American Association for Clinical Chemistry [41], we performed a sensitivity analysis considering semi-quantitative Ct values. The levels of Ct can be separated into three groups, according to Quiroz-Ruiz and co-authors [42]. These authors proposed considering the clinical severity and the Ct value: minor (Ct < 18.83), medium (Ct ≥ 18.83–30.10), and high (Ct > 30.10). Multinomial logistic regression analysis was performed considering crude and adjusted odds ratios (OR) with potential confounding factors, as indicated before.

For this study, the approval of an Ethics Committee was not needed considering the epidemic situation of the COVID-19 pandemic in accordance with Spanish legislation and regulations about the epidemiological surveillance of COVID-19 outbreaks, including the General Law of Health [43], the Law of Cohesion and Quality of the National System of Health [44], the Law General of Public Health [45], and the Early Response Plan in a COVID-19 Pandemic Control Scenario [46]. In addition, informed consent was not required from the participants as this was a retrospective study without the names of the participants.

## 3. Results

Nursing home characteristics, SARS-CoV-2 infections, and reinfections are shown in Table 1. The SARS-CoV-2 variants of the four outbreaks were Delta B.1.617.2, one outbreak in April 2021, and Omicron, three from January to March 2022. The four nursing homes had a total of 472 residents; the mean age was 83.1 ± 9.8 years with 318 females (67.4%), and 467 were vaccinated with two doses of the SARS-CoV-2 vaccine (98.9%). The median of the elapsed time from the former SARS-CoV-2 outbreak was 391.8 days, with a range of 339.9 to 634.9 days. In the four nursing homes, the means attack rates of infections and reinfections were 72.7% (range 45.6–96.2%) and 19.9% (range 0.9–47.7%), respectively, and the means mortality and case fatality rates were 4.0% and 5.5%, respectively.

**Figure 1:**
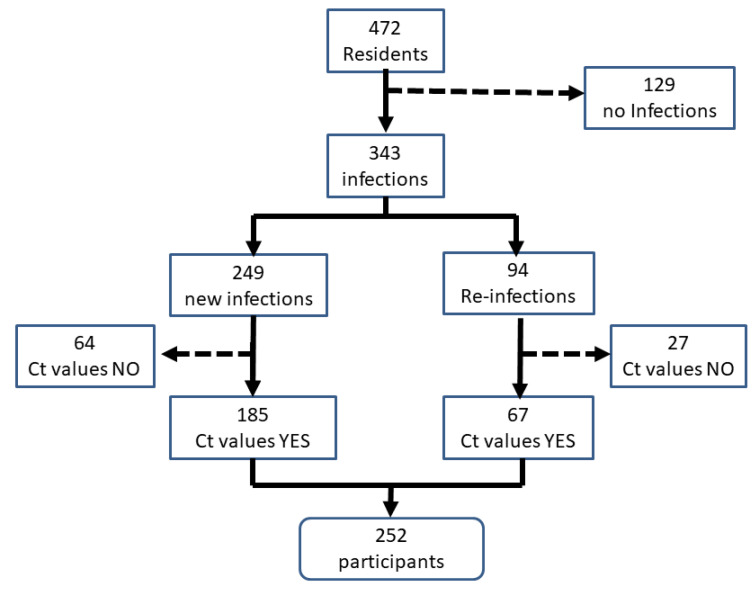
Flow diagram showing the residents of nursing homes participants with cycle threshold values (Ct) in the study.

Considering the 472 residents, a total of 343 residents suffered SARS-CoV-2 new infections (Figure 1). From them, 249 first infections and 94 reinfections were reported. Ct values determinations were made for 252 residents, 185 with new infections and 67 with reinfections, with a participation rate of 73.5%.

The characteristics of participants and Ct values of SARS-CoV-2 infections and reinfections are shown in Table 2. The mean of reinfections was 26.6% (range 1.1–53.3%). When comparing Ct at each nursing home, the mean Ct values were 28.4 ± 6.7 (range 25.2 ± 6.2–30.9 ± 7.0). The Ct mean in reinfection of residents was 31.9 ± 5.4 versus 27.1 ± 6.6 in residents with the first infections (*p* = 0.000). With respect to quantitative Ct values, in a crude analysis of robust bivariate linear regression, the regression coefficient was 4.75 (95% CI 2.97–6.52, *p* = 0.000) and in a robust multivariable linear regression, it was 4.78 (95% CI 2.80–6.76, *p* = 0.000).

A comparison of Ct values ≥ 30 versus <30 by Poisson regression is presented in Table 3. In the adjusted analysis, the elapsed time from the former SARS-CoV-2 outbreak was significantly less in the group with Ct values < 30. In three nursing homes, patients with SRAR-CoV-2 reinfections had a higher Ct than patients with the first infection, but only in a center with a significant difference. In the four nursing homes, patients with reinfections had higher Ct values than patients with the first infection, adjusted relative risk 1.66 (96% CI 1.10–2.51, *p* = 0.015).

The results of the sensitivity analysis are shown in Table 4. Patients with SARS-CoV-2 reinfections had significantly more elevated Ct values than patients with new SARS-CoV-2 infection, adjusted odds ratio of 17.63 (95% CI 2.22–139.86, *p* = 0.007) for Ct values > 30.1 with respect to Ct values < 18.83.

## 4. Discussion

Our results suggest the usefulness of Ct values in SARS-CoV-2 outbreaks in nursing homes for knowing the epidemic’s evolution and being an appropriate surveillance tool in outbreaks. The Ct values have increased in SARS-CoV-2 reinfections, and the epidemic situation has decreased with less transmission and severity, following the paradigm of hybrid immunity [47,48]. The mortality and case fatality was considerably reduced in the four nursing homes compared with the first COVID-19 epidemic from March 2020 to January 2021 in the nursing homes in the Castellon Health Department, when mortality and case fatality means were 8.7% and 22.7%, respectively [17]. Although there were elevated proportions of SARS-CoV-2 vaccinated residents, the incidence of SARS-CoV-2 infections was high, suggesting that vaccination has limited efficacy against viral transmission, and non-pharmacological measures in these nursing homes need to be improved to reduce transmission of SARS-CoV-2 infections [49,50].

In three nursing homes, Ct values were associated with SARS-CoV-2 reinfection, except in the center, where the elapsed time from the former SARS-CoV-2 outbreak was longer than one year and a half. Anti-SARS-CoV-2 antibodies decline over time after infection or vaccination [51], and the study of cellular immunity in nursing home residents could be useful [52].

In a SARS-CoV-2 outbreak, a serial of Ct values can provide information on the clinical and epidemiological situation of residents and staff and take adequate measures of control and prevention, such as non-pharmaceutical interventions to prevent transmission [53]. In Massachusetts, Ct values for staff and residents of nursing homes had no differences between symptomatic and asymptomatic patients at the time of sampling, with mean Ct values of 25.7 and 26.4, respectively [54]. During a SARS-CoV-2 outbreak in a nursing home in Holland, Paad and co-authors [55] reported similar findings in residents. However, Wilson and co-authors [56] found that residents and healthcare personnel in nursing homes with specimen Ct < 30 were more likely to have symptoms, and only 17% of positive SARS-CoV-2 individuals after more than 90 days of the first infection had Ct values less than 30. Testing for infectivity should be performed without considering symptoms of infection. In addition, SARS-CoV-2 vaccines (ChAdOx1 nCoV-19 and BNT162b2) one or two doses can increase the Ct values of vaccinated residents in nursing homes compared with no vaccinated residents and suggest that the vaccine may protect against virus transmission [21]. Shrotri and co-authors found [57] that the mean of Ct values was higher for infection after vaccination than for infection before vaccination (31.3% versus 26.6%, *p* < 0.0001).

At a population level, routine screening of Ct values can be useful for monitoring the SARS-CoV-2 epidemic [58]. In the general population of Pakistan, Shoaib and co-authors [59] found an increase in Ct values in asymptomatic cases of SARS-CoV-2 to decrease epidemic possibility. In Iran, Dehesh and co-authors [60] found that the average daily Ct value can predict increases in the number of positive confirmed COVID-19 cases. In England during the period 2020–2022, Harrison and co-authors [15] found that mean Ct values decreased 6–29 days before the number of positive tests increased, and Ct values provided an indication of new variants. In Delta variant outbreaks, Ct values were a predictor for hospitalization in Belgium [61]. Measures of Ct values for future SARS-CoV-2 epidemics have been highlighted using the third generation of RT-PCR [62]. In addition, a method of standardization of Ct values in the function of the clinical sample has been proposed [63,64].

The strengths of this study include: first, a cohort design that reduces bias in selection and information. Second, the participation rate was elevated. Third, confirmatory tests were completed for all the residents. Fourth, only two laboratories with the same RT-PCR technique carried out all the samples. Fifth, multivariable models were used to control confounding factors. Sixth, the sensitivity analysis confirms the results.

The RT-PCR is a qualitative test, and its use as a quantitative test or semi-quantitative test is the more important limitation of our study [27,41,65,66,67]. More limitations include: First, the Ct groups used in our study are based on clinical severity [68]. Second, Ct values present changes associated with the duration of the illness, increasing with the elapsed time from the onset of the illness [18,69]. Third, during an outbreak, the timing of sampling could present a mix of different durations of the disease. Fourth, in the comparisons of Ct values among laboratories, differences could occur considering the specific work of each laboratory, such as expertise and protocols used [18,28,70].

Considering the hypothesis of our research and corroboratory results, the implementation of surveillance of SARS-CoV-2 infections in nursing homes by the measure of Ct values could be useful for monitoring outbreaks` dynamics, virus transmission, and the adequacy of preventive measures. Future prospective cohort studies in nursing homes could be performed in order to prove the applicability and relevance of this approach.

## 5. Conclusions

In our cohort study with a high proportion of SARS-CoV-2 vaccinated residents, reinfections were associated with high Ct values that correspond with hybrid immunity, suggesting a decrease in transmission and severity, and can be a tool of epidemiological surveillance of SARS-CoV-2 epidemics in nursing homes.

## Figures and Tables

**Table 1:** Description of nursing homes SARS-CoV-2 outbreaks, dates, SARS-CoV-2 variants, demographic characteristics, SARS-CoV-2 vaccination levels, and SARS-CoV-2 infections, reinfections, and case fatality rates.

Variables	Center 1 n (%)	Center 2 n (%)	Center 3 n (%)	Center 4 n (%)	Total n (%)
Date of reporting (month/year)	August 2021	March 2022	January 2022	February 2022	
COVID-19 Variant outbreak	Delta B.1.617.2	Omicron	Omicron	Omicron	
Total residents	125	130	111	106	472
Age (years) mean SD ^1^	84.4 ± 8.3	82.0 ± 11.0	83.8 ± 8.9	82.4 ± 10.8	83.1 ± 9.8
Female	90 (72%)	92 (70.8%)	74 (66.7%)	62 (58.5%)	318 (67.4%)
Male	35 (28%)	38 (29.2%)	37 (33.3%)	44 (41.5%)	154 (32.6%)
Elapsed time from former SARS-CoV-2 outbreak in days	339.9	433.1	350.5	634.9	391.8 ^2^
SARS-CoV-2 Vaccine	122 (97.6%)	130 (100%)	111 (100%)	104 (98.1%)	467 (98.9%)
Elapsed time from the last vaccine doses; mean SD in days	177 ± 28.4	154 ± 73.6	108 ± 36.2	154 ± 22.2	157 ± 50.1
Total SARS-CoV-2 infections	57 (45.6%)	125 (96.2%)	94 (84.7%)	67 (63.2%)	343 (72.7%)
Incidence attack rate (%) ^3^	45.6%	96.2%	84.7%	63.2%	72.7%
New SARS-CoV-2 infections	50 (40%)	63 (48.5%)	93 (83.8%)	43 (40.6%)	249 (52.8%)
SARS-CoV-2 reinfections	7 (5.6%)	62 (47.7%)	1 (0.90%)	24 (22.6%)	94 (19.9%)
Reinfections attack rate (%) ^3^	5.6%	47.7%	0.90%	22.6%	19.9%
Mortality rate	6 (4.8%)	7 (5.4%)	6 (5.4%)	0	19 (4.0%)
Fatality rate	10.6%	17.9%	1.1%	0	5.5%

^1^ SD = standard deviation. ^2^ Median. ^3^ On the total of residents in each center.

**Table 2:** Characteristics of residents with SARS-CoV-2 infections and reinfections and cycle threshold values (Ct) in nursing homes.

Variables	Centre 1 n (%)	Centre 2 n (%)	Centre 3 n (%)	Centre 4 n (%)	Total n (%)
Residents with PCR-Ct	38	90	92	32	252
Age	85.0 ± 6.7	81.9 ± 11.6	84.7 ± 8.4	80.4 ± 12.0	83.2 ± 10.0
Female	32 (84.2)	66 (73.3)	66 (71.7)	20 (62.5)	184 (73.0)
Male	6 (15.8)	24 (26.7)	26 (28.3)	12 (37.5)	68 (27.0)
First infection with PCR-Ct	33 (86.8)	42 (46.7)	91 (98.9)	19 (59.4)	185 (73.4)
Reinfections with PCR-Ct	5 (13.2)	48 (53.3)	1 (1.1)	13 (40.6)	67 (26.6)
Reinfections without PCR-Ct	2 (5.3)	14 (15.6)	0	11 (34.4)	27 (10.7)
Cycle threshold (median range)	23.3 (17–38)	33.5 (11–39)	27 (17–38)	29 (15–37)	29 (11–39)
Cycle thresholds’ mean ± SD	25.2 ± 6.2	30.9 ± 7.0	27.2 ± 5.7	28.4 ± 6.6	28.4 ± 6.7
Ct SARS-CoV-2 reinfections	36.4 ± 3.0	32.5 ± 4.9	26.0	28.1 ± 5.7	31.9 ± 5.4
Ct first SARS-CoV-2 infections	23.5 ± 4.5	29.0 ± 8.5	27.3 ± 5.7	28.6 ± 7.3	27.1 ± 6.6
*p*-values	0.000	0.016	0.876	0.820	0.000

**Table 3:** Comparison of cycle thresholds ≥30 versus <30 for demographic, elapsed time from the former SARS-CoV-2 outbreak, nursing homes, and first SARS-CoV-2 infections and reinfections by Poisson regression. Crude and adjusted relative risk (cRR) and (aRR) 95% Confidence Interval (CI).

Variables	Ct ≥ 30 n = 123 (%)	Ct < 30 n = 129 (%)	Total	cRR	95% CI	aRR	95% CI	*p*-Value
Age ^1^ mean ± SD	82.9 ± 10.1	83.5 ± 10.0	-	1.00	0.98–1.01	0.99 ^1^	0.98–1.01	0.675
Male ^2^	32 (47.1)	36 (52.9)	68	0.95	0.64–1.42	0.92 ^2^	0.59–1.42	0.703
Female	91 (49.5)	93 (50.5)	184	1.00		1.00		
Elapse time from former SARS-CoV-2 outbreak ^3^ (days) mean ± SD	427.9 ± 86.8	401.7 ± 97.3	-	1.00	0.99–1.00	1.00 ^3^	1.00–1.01	0.031
Nursing homes ^4^								
Centre 1								
SARS-CoV-2 Reinfections	5 (100)	0 (0)	5	6.60	1.91–22.80	6.70	1.80–25.0	0.005
First SARS-CoV-2 Infections	5 (15.2)	28 (84.8)	33	1.00				
Centre 2								
SARS-CoV-2 Reinfections	39 (81.3)	9 (18.8)	48	1.31	0.80–2.16	1.31	0.80–2.15	0.287
First SARS-CoV-2 Infections	26 (61.9)	16 (38.1)	42	1.00				
Centre 3								
SARS-CoV-2 Reinfections	0 (0)	1 (100)	1	1.93 ^5^	0.0–10.76	2.18 ^6^	0.0–14.25	1.000
First SARS-CoV-2 Infections	33 (34.1)	58 (63.7)	91	1.00				
Centre 4								
SARS-CoV-2 Reinfections	5 (38.5)	8 (61.5)	13	0.73	0.20–2.34	0.70	0.24–2.05	0.513
First SARS-CoV-2 Infections	10 (52.6)	9 (47.4)	19	1.00				
Total nursing homes ^7^								
SARS-CoV-2 Reinfections	49 (73.1)	18 (26.9)	67	1.83	1.27–2.62	1.66	1.10–2.51	0.015
First SARS-CoV-2 Infections	74 (40.0)	111 (60.0)	185	1.00		1.00		

^1^ Adjusted for sex. ^2^ Adjusted for age. ^3^ Adjusted for age, sex and nursing homes. ^4^ Adjusted for age and sex. ^5^ Exact Poisson regression. ^6^ Exact Poisson regression. ^7^ Adjusted for age sex elapsed time from former SARS-CoV-2 outbreak and nursing homes.

**Table 4:** Sensitivity analysis: multinomial logistic regression. Cycle threshold and reinfections. Crude and adjusted odds ratios (cOR) and (aOR) 95% confidence interval (CI).

	Yes n = 67	No n = 185	cOR	95% CI	aOR	95%	*p*-Value
Cycle threshold ^1^	n (%)	n (%)					
Minor (<18.83)	1 (1.5)	19 (10.3)	1.00		1.00		
Medium (≥18.83–30.10)	17 (25.4)	99 (53.5)	3.26	0.41–26.00	5.98	0.73–49.12	0.096
High (>30.10)	49 (73.1)	67 (36.2)	13.90	1.80–107.33	17.63	2.22–139.86	0.007

^1^ Adjusted age sex elapsed time from former SARS-CoV-2 outbreak nursing homes.

## Data Availability

Authorization of the Public Health Center’s direction will be required to consult the data set of this study.
